# A Study of Early Afterdepolarizations in a Model for Human Ventricular Tissue

**DOI:** 10.1371/journal.pone.0084595

**Published:** 2014-01-10

**Authors:** Nele Vandersickel, Ivan V. Kazbanov, Anita Nuitermans, Louis D. Weise, Rahul Pandit, Alexander V. Panfilov

**Affiliations:** 1 Department of Physics and Astronomy, Ghent University, Ghent, Belgium; 2 Department of Theoretical Biology, Utrecht University, Utrecht, The Netherlands; 3 Center for Condensed Matter Theory - Department of Physics, Indian Institute of Science, Bangalore, India; Georgia State University, United States of America

## Abstract

Sudden cardiac death is often caused by cardiac arrhythmias. Recently, special attention has been given to a certain arrhythmogenic condition, the long-QT syndrome, which occurs as a result of genetic mutations or drug toxicity. The underlying mechanisms of arrhythmias, caused by the long-QT syndrome, are not fully understood. However, arrhythmias are often connected to special excitations of cardiac cells, called early afterdepolarizations (EADs), which are depolarizations during the repolarizing phase of the action potential. So far, EADs have been studied mainly in isolated cardiac cells. However, the question on how EADs at the single-cell level can result in fibrillation at the tissue level, especially in human cell models, has not been widely studied yet. In this paper, we study wave patterns that result from single-cell EAD dynamics in a mathematical model for human ventricular cardiac tissue. We induce EADs by modeling experimental conditions which have been shown to evoke EADs at a single-cell level: by an increase of L-type Ca currents and a decrease of the delayed rectifier potassium currents. We show that, at the tissue level and depending on these parameters, three types of abnormal wave patterns emerge. We classify them into two types of spiral fibrillation and one type of oscillatory dynamics. Moreover, we find that the emergent wave patterns can be driven by calcium or sodium currents and we find phase waves in the oscillatory excitation regime. From our simulations we predict that arrhythmias caused by EADs can occur during normal wave propagation and do not require tissue heterogeneities. Experimental verification of our results is possible for experiments at the cell-culture level, where EADs can be induced by an increase of the L-type calcium conductance and by the application of I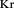 blockers, and the properties of the emergent patterns can be studied by optical mapping of the voltage and calcium.

## Introduction

The mechanical pumping of the heart is initiated by electrical waves of excitation. The abnormal propagation of such waves may result in cardiac arrhythmias which disrupt the normal pattern of cardiac contraction and can, therefore cause cardiac arrest and sudden cardiac death [1]. Thus, understanding the mechanisms of initiation of cardiac arrhythmias is of great importance for practical cardiology. Unfortunately, we do not know yet the exact mechanisms by which such arrhythmias occur in the human heart, however, several factors have been shown to be correlated with increases in the incidence of arrhythmias. One such factor is the onset of excitations of cardiac cells with an abnormal time course of the action potential (AP), such as early after depolarizations (EADs) [2]–[4]. In general, an EAD is defined as a reversal of the action potential before the completion of its repolarization. It can occur in many forms of genetic defects such as the long QT syndrome [5]–[7], under the action of pharmacological agents as a result of cardiotoxicity [8], [9], and in several other conditions [10], [11]. Such EADs are often related to cardiac arrhythmias like Torsades de Pointes (TdP) [12]–[14]. However, the mechanisms of how abnormal excitations result in arrhythmias is still a widely studied subject because many questions remain unanswered.

One of the challenges in the study of EADs is that they occur normally at the level of a single-cell, as a result of mutations or changes of properties of the individual ionic channels. Therefore, the first question is to find the conditions that are responsible for the onset of EADs. An action potential is generated by many different interacting ionic channels, hence, gradual controlled changes of the properties of an individual channel and studies of their effects on the action potential is a non-trivial problem for experimental research. The second important question is to find mechanisms for the progression of EADs to cardiac arrhythmias. Although EADs occur at a single-cell level, cardiac arrhythmias occur because of abnormal wave propagation at the tissue or whole-heart level. The relation of such abnormal propagation to single-cell behaviors is a complex problem. The solution of such a challenging problem requires that we complement experimental studies by alternative methods. One such method is multi-scale mathematical modeling, which is now widely used in studies of cardiac arrhythmias [15]–[18].

The first question, namely, how changes in ionic currents may result in EADs, was addressed in earlier modeling studies. The first studies were performed in the group of Rudy [19]–[21] by using a guinea-pig-cell model. They investigated the mechanism of the generation of EADs and their rate dependence, and they made the link to the long-QT syndrome. Later, in Ref. [22], a two-parameter region of the existence of EADs was determined in the LR1 model [23] by changing the maximal conductance of the L-type Ca-channels and the time constant of the 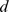 gate. In studies of Ref. [24], the generation of EADs was investigated by invoking the LQT3 syndrome, followed by mild hypokalaemia and a partially blocked delayed rectifier current 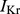. In all these computational studies, EADs were obtained by altering I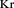, I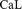, the L-type Ca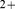 current, I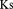, the slow delayed rectifier current, or I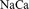, the Na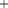/K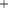 exchanger current. In many cases such changes were made to mimic the onset of the long-QT syndrome or to describe the action of a certain drug. Except in the study of Ref. [22], only a few parameters were changed and then set to very specific values. Comprehensive studies, which require gradual changes of parameters and investigations of parameter ranges, when changing the currents, have not been attempted. Moreover, all these studies have used mathematical models for animal cardiac cells and not for human cardiac cells.

The second question, namely, how EADs can progress to cardiac arrhythmias, has been studied much less than the first one. Reference [14] has addressed the problem of how individual-cell events can cause wave initiation. It has shown that the generation of EADs has all features of a chaotic process, and that such chaotic behavior can synchronize spatially to overcome source-sink mismatches and form propagating waves. This study has also provided examples of the formation of complex spatiotemporal patterns in a rabbit-tissue model [25]. Similarly, in [26] they have also studied how single-cell EADs synchronize to result in two-dimensional (2D) patterns, here in heterogeneous tissue. In Ref. [27], male- and female-hormone effects have been studied in a guinea-pig model of cardiac tissue. This study has demonstrated EAD formation in the late-follicular phase and has elucidated 2D wave propagation under such conditions. It has shown, furthermore, that, in a simulated-wedge preparation, EADs can generate arrhythmia sources, as it creates local heterogeneity in the refractory period. In Ref. [28], a bistable, i.e., 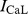 and 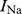, wave propagation has been found in computational and experimental studies by using EAD-sensitive cell preparations. Furthermore, complex wave dynamics, including the meandering of an 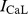-mediated spiral wave in heterogeneous tissue, has been obtained. In summary, although some papers have shown examples of complex wave patterns in 2D EAD preparations, there has been no systematic study that explores the relation of single-cell EAD excitations and their 2D manifestations. In addition, only mathematical models for animal cardiac cells have been used in such studies so far. Therefore, the aim of our paper is to perform a systematic study of EAD-induced, 2D, excitation patterns by using the TNNP-TP06 mathematical model for human ventricular tissue [29], [30].

Our study yields several new and interesting results, which we summarize here before we present the details of our work. We first find regions of existence of EADs in a single-cell in a modified TNNP-TP06 model and obtain three two-parameter portraits of their existence by varying 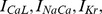 and 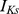. As in Ref. [31], we classify the resulting action potentials (APs) into normal APs, APs with EADs, and nonrepolarizing APs or oscillatory APs. Furthermore, we study 2D excitation patterns, originating from each of the EAD types, by using two different kinds of initial conditions. Our simulations show that the observed spatial patterns can be divided into 3 classes, which are characterized by different degrees of complexity and that may also include exotic regimes, such as those with phase-wave propagation. We demonstrate that the existence of EAD patterns, at the cellular level, is a good predictor for chaotic spatial patterns of excitation in 2D domains. We show, finally, that a decrease of repolarization reserve progressively changes the spatial patterns, in our 2D simulations, from chaotic spirals to regular Ca-spirals, and finally to ones that have spatial oscillations. We also discuss the importance of our results for EAD arrhythmias and discuss possible future experiments that could be designed to test the results of our study.

## Materials and Methods

### Model for Human Ventricular Tissue

In this paper, we use the recent TNNP-TP06 model for human ventrical cells [29], [30]. For a single-cell, this model is defined by the ordinary differential equation:
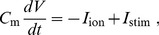
(1)where 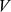 is the voltage that describes the action potential, 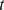 is the time, 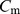 is the cell capacitance per unit surface area, 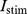 is the externally applied stimulus, and 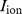 is the sum of the following ionic currents:
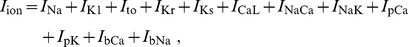
(2)where I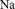 is the Sodium current, I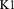 is the inward rectifier K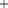 current, I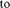 is the transient outward current, I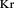 is the delayed rectifier current, I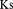 is the slow delayed rectifier current, I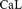 is the L-type Ca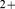 current, I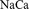 is the Na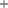/K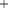 exchanger current, I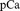 and I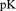 are plateau Ca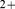 and K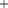 currents, and I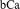 and I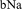 are background Ca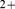 and K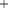 currents. The precise behavior of the different channels is based on a wide range a human-based electrophysiological data, and details can be found in Ref. [29], [30]. Below, we provide a description of a few ionic currents that are important for our study.

The L-type Calcium current is given by
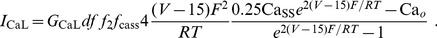
(3)


Here 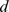, 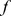, 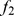 and 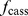 represent the gating variables: 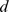 is a voltage-dependent activation gate, 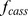 is a fast-subspace calcium-inactivation gate, 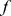 is a slow, voltage-inactivation gate, and 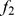 is a fast, voltage-inactivating gate. Fast and slow gates are distinguished by their times constants, in particular, 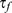 represents the time constant of the 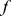 gate. We have made a few modifications in 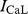, compared to its form in the original TNNP-TP06 model. In particular, to obtain EADs, we have decreased twofold the time constant 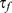 (see also Ref. [19]). As this results in some shortening of the AP, we have increased the value of 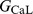 by a factor of 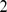. This modified version of the TNNP-TP06 model gives almost the same representation of the restitution properties of the cell, as in the original TNNP-TP06 model, in the most important interval of external stimulation (with the period 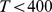 ms). We refer to Table 1 for the value of 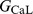. For completeness, 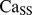 is the concentration of 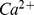 in the subspace, and 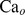 is the extracellular 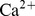 concentration. 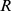, 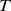 and 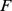 represent the gas constant, the temperature (310 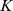) and the Faraday constant.

**Table 1:** Maximal conductances of the relevant channels

Parameter	Value	Current
G 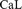	2 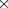 0.00003980 mm 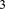 /(ms 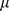 F)	I 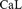
G 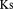	0.3923027 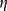 S/pF	I 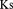
G 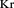	0.1532432 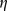 S/pF	I 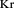
k 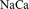	1000 mV/ms	I 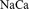

In this table, we show the maximal conductances of the ionic channels which were altered in this paper. In “current”, one can find the corresponding current of this conductance. Notice that G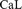 is increased twice in comparison with [30].

The slow delayed rectifier current 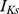 is given by,
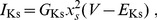
(4)where 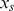 is an activation gate and E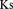 is a reversal potential [29]. These channels can be blocked by altering the maximal conductance G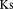, see Table 1.

Next, we have the rapid, delayed rectifier current
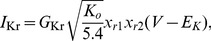
(5)where 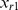 is an activation gate, 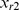 is an inactivation gate, 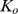 is the extracellular K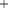 concentration, and E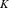 is some reversal potential [29]. 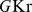 is the maximal conductance, see Table 1.

Another important current in our simulations is the 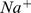/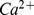 exchanger current
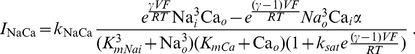
(6)


Here, 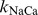 is the maximal channel conductance, see 1, 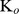 and 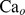 are the extracellular K and Ca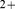 concentration, Na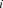 and Ca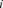 are intracellular concentrations. Finally, for completeness, we also include 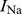,
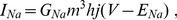
(7)where 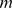 is an activation gate, 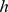 is a fast inactivation gate, 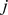 is a slow inactivation gate, and 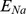 is the reversal potential. As other parameters are not directly relevant for our paper, we refer the reader for more details on these parameters and for a descriptions of all currents to Refs. [29], [30].

For completeness, in Table 1, we give an overview of all the values used of all the maximal channel conductances mentioned in this paper.

At the tissue level we used a standard mono-domain description for isotropic cardiac tissue:
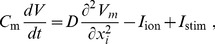
(8)with 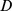 the diffusion coefficient, and 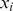, 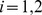, the 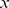 and 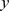 coordinates.

For a more detailed description of the model, we refer to [29] and [30]. All default parameters were taken from these papers. Because many of the experimental measurements were performed for endocardial cells, see e.g. [10], we use an endocardial cell parameter set of the TNNP-TP06 model.

### Numerical methods

In order to obtain an EAD, during the plateau phase of the action potential, the inward currents should exceed the outward currents. Therefore, we alter the conductances of the different ion channels, such as those associated with the currents 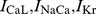 and 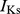. In our single-cell simulations, we stimulate the cardiac cells by applying a current of 20 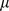 A/mm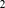 for 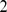 ms.

**Figure 1:**
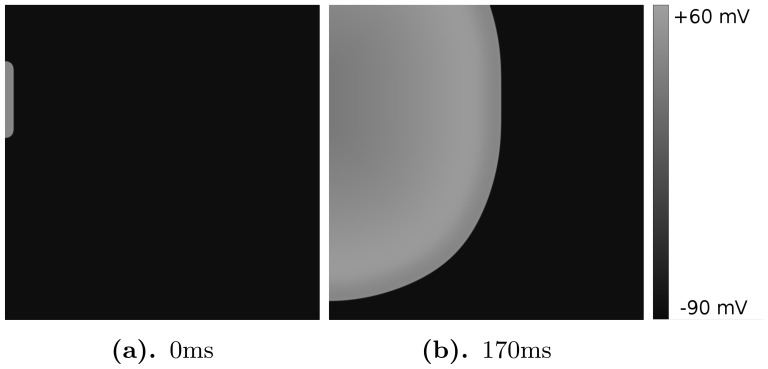
Protocol P1. A wave is initiated at the left of the tissue by stimulating a region of 6 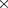 200 points, located at the left boundary and propagates over the entire tissue.

**Figure 2:**
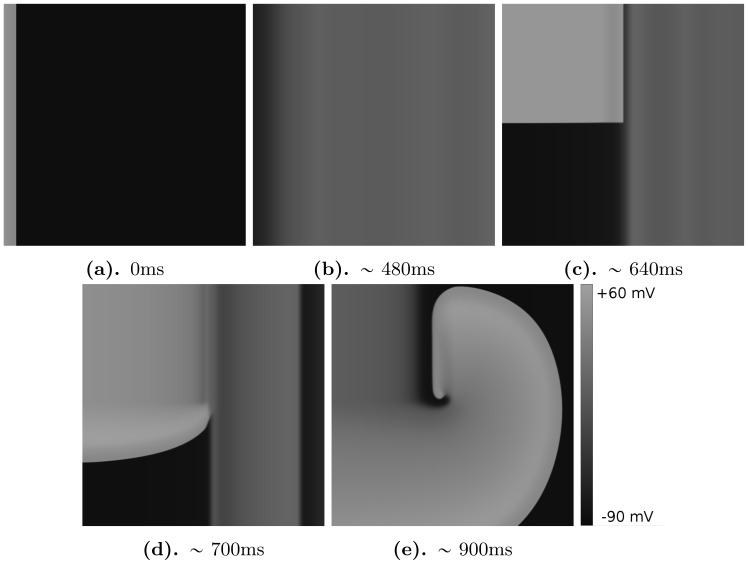
Protocol P2. We induce a spiral wave by using the standard S1–S2 stimulation protocol. We first stimulated the left side of the domain (a), as in protocol P1, to induce a plane wave that propagates from the left side of the domain to its right boundary (b). Once this wave has passed over the first half of the domain, we applied a second stimulus in the first quarter of the domain (c), which induces the spiral (d)–(e).

All our two-dimensional (2D) simulations have been carried out in an isotropic domain with 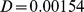 cm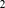/ms, with a time step of 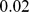 ms and a space step of 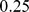 mm in both 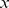 and 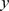 directions. These 2D simulations have been performed on a domain of 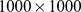 grid points by using the explicit-Euler integration scheme. We have used Neumann (i.e., no-flux) boundary conditions, and a 5-point stencil for the Laplacian. We use two different types of initial conditions, which we have generated by the following 2 protocols. In the first protocol, P1, we have stimulated a region of 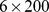 points, located at the left boundary, by a current of 20 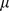 A/mm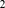. for 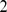 ms, as illustrated by the gray-scale plot of the transmembrane potential 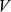 in Figure 1. In the second protocol, P2, we have induced a spiral wave by using the standard S1–S2 stimulation protocol [32]. Here, we first stimulated the left side of the domain, as in protocol P1, to induce a plane wave that propagates from the left side of the domain to its right boundary. Once this wave has passed over the first half of the domain, we apply a second stimulus in the first quarter of the domain. This induces the formation of a spiral wave, which we show in the gray-scale plot of 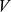 in Figure 2.

## Results

We present the results of our extensive numerical studies here. We begin with single-cell studies and then discuss the results of our 2D simulations.

### Single-cell

#### Phase diagrams for single-cell simulations

**Figure 3:**
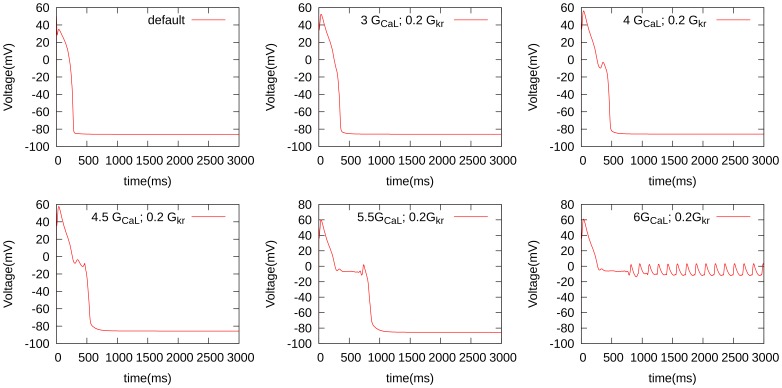
The development of EADs for different parameters. The channel conductivity of the L-type Ca is enhanced, while the slow delayed rectifier channel conductivity is reduced as indicated in the figures.

**Figure 4:**
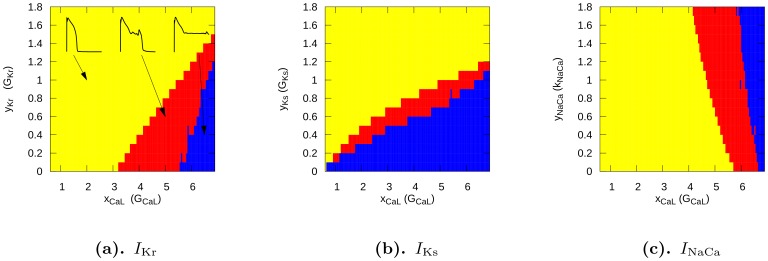
Parametric space of different AP behaviour. The numbers on the 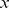 axis give the multiplication factor for the maximal calcium conductance, the 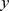 axis for the maximal conductance of (a) 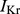 (b) 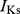 (c) 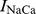. Yellow, red, and blue colors indicate, respectively, regions in which the AP has no EAD, the AP appears with EADs, and the AP does not return to the resting potential.

As EADs occur because of an increase in inward currents and/or a decrease in the outward current, we have first performed studies in which we progressively increased 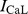 and decreased one of the potassium currents (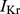 or 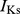). Figure 3 shows the shape of the AP and how it changes as we increase 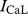, for 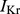 at 20% of its maximal value. We see, in Figure 3, that, with 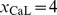 (i.e., a 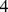-fold increase of the Ca conductance), we get a single-EAD response. For larger values of 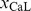, we see multiple and more complex AP shapes, and for 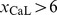, we obtain oscillatory AP dynamics. We have performed similar studies for several sets of two-parameter changes, the results are shown in Figure 4. To represent them, we have subdivided all possible AP shapes into the following 3 types: normal APs, APs with one or more EADs, and oscillatory APs. In all these cases we find, in consonance with our expectations, that increases in 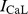 or 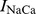 and decreases in 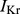 or 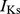 promote transition from normal APs to those with EADs and to APs with oscillations. These findings are in line with those in Ref. [22]. We can obtain EADs in a fairly large parameter regime, when we allow for changes of 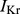 and 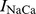, and in a slightly smaller region, when we block 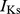. All our figures show similar dynamics under the parameter changes mentioned above. Therefore, we have only used the parameter set of Figure 4(a) in our 2D studies. Before we present our 2D results, we consider briefly the mechanisms of EADs in our model.

### Mechanism of the generation of EADs

**Figure 5:**
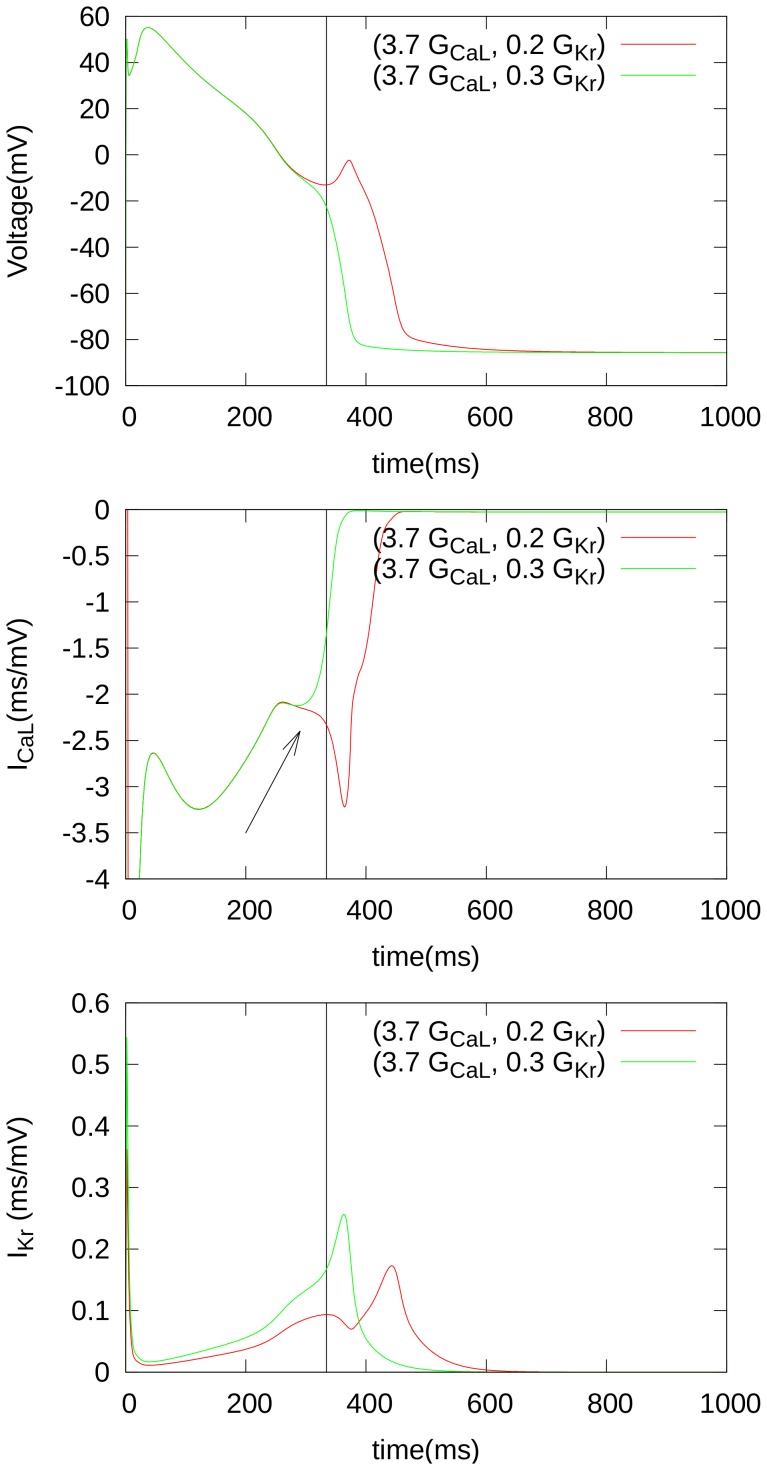
Important ionic currents related with EAD formation. The parameters of the red curves are 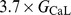, 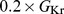 (EAD), while the parameters of the green curves are 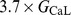, 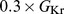
(no EAD).

Figure 5 shows, for two parameter values, APs just before (green curve) and just after (red curve) EAD formation. We see that the main driving current for EADs is 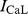, i.e. its re-activation causes extra depolarization. However, for this depolarization to occur, the value of 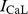, at a given moment, should exceed the value of the outward currents, as we see in the bottom panel of Figure 5, where we depict the difference between 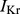 in the green and red curves. Such a decrease of 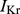 occurs in the vicinities of point where EAD formation starts. For an overview of all the currents, we refer the reader to the Figure S1 in the Supplementary Material. This figure shows, indeed, that two currents are responsible for the formation of the EAD, namely 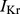 and 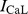, which are exactly the currents that we have altered in our simulations.

In Figure S2, see the Supplementary Material, we also see that, because of the elevation of the L-type Ca current, the ryanodine receptors can be triggered. This results in calcium-induced calcium release (CICR) and the subsequent activation of I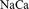 and is similar to the mechanisms discussed in Ref. [11]. However, this is a secondary effect, and not a trigger for EAD formation. Moreover, there are cases in which EADs occur without CICR, e.g. see also Figure S2.

1 For other parameter values, we obtain qualitatively similar results.

### 2D simulations

#### Phase diagrams for 2D simulations

To address the important issue of the manifestations of the aforementioned single-cell patterns in 2D tissue, we have performed extensive, 2D simulations by using initial-point stimulation (protocol P1, Figure 1) and S1–S2 stimulation (protocol P2, Figure 2). We have given the details of these protocols in the Methods Section. The idea of these two different protocols was to target two processes: initiation of the first spiral and thus the onset of arrhythmias and a possible breakup of a spiral into a comples statio temporal pattern or thus transition from tachycardia to fibrillation. Although these processes are similar they are not identical and may well be induced by different mechanisms.

**Figure 6:**
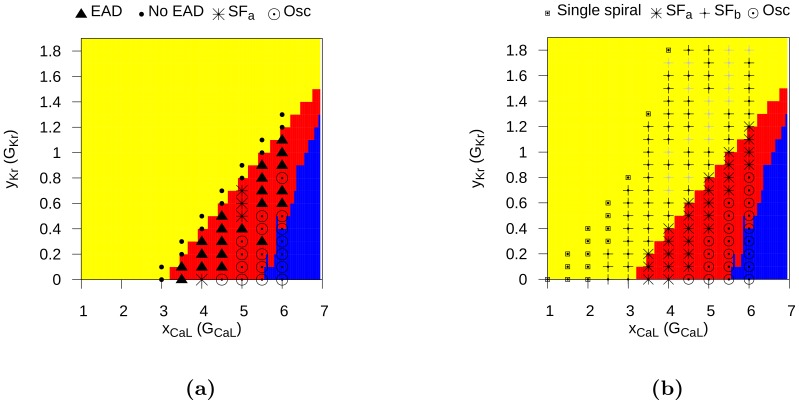
Parametric space of EADs caused spatial patterning. This figure shows a phase diagrams, in a two-dimensional parameter space, of the different types of excitation patterns, obtained by using the stimulation protocols (a) P1 (56 simulations) and (b) P2 (134 simulations). The yellow, blue, and red colors indicate, respectively, no EAD, EAD, and oscillatory AP single-cell behaviors, as in Figure 4a. The symbols are as follows: SF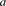 (*), SF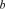 (+), oscillatory fibrillation (⊙), wave propagation without EAD (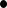), wave propagation, in which the AP shows EADs, but there is no sustained electrical activity (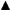), and a typical non-EAD pattern with a stable, single spiral wave (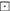). The light-gray SF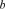 represents SF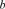-type patterns which terminate themselves during our simulations, which happened in the first stages of the break up of the spiral.

**Figure 7:**
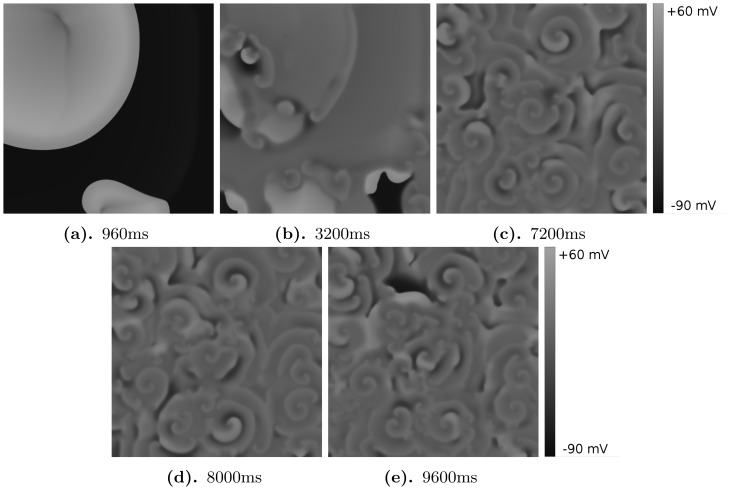
Illustration of spontaneous spiral fibrillation of type a. Protocol 1 is used with parameters 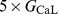, 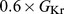.

**Figure 8:**
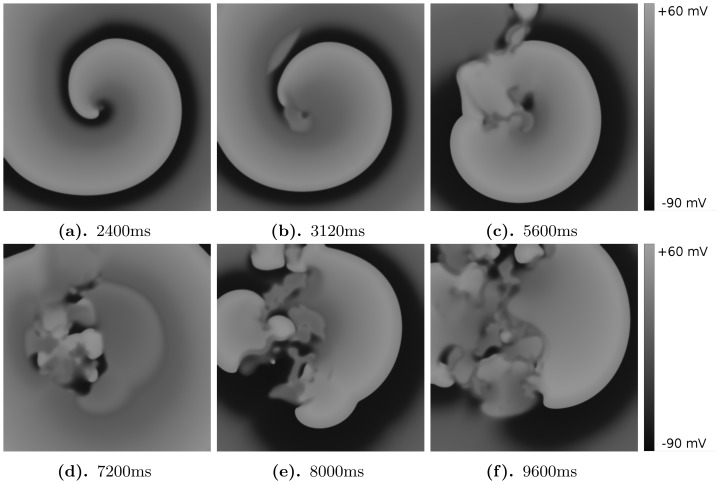
Illustration of spiral fibrillation type b. Protocal 2 is used with parameters 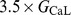, 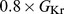.

Our study leads to a classification of excitation patterns into the following three different types, see Figure 6: (a) spiral fibrillation, type a (SF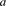: 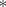), (b) spiral fibrillation, type b (SF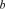: (+)), and (c) oscillatory fibrillation (⊙). The first two patterns are formed due to breakup of the wave, which eventually lead to the final patterns SF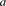 and SF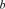. The first final pattern SF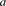 consist of many small rotating spirals, as shown in Figure 7, and it shows almost no black regions, which means that the cells do not completely return to the resting state. Later on, we show that these patterns consist of Ca-waves. The second final pattern SF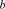 can be found in Figure 8, which shows a chaotic pattern of waves that do not show clear rotations anymore. Also, many more black regions can be observed. Later we show that these patterns consist of Na-waves. The third oscillatory pattern consist of individual cells which all oscillate but synchronize, so it seems that they produced waves. Later, however, we show that these waves are in fact phase waves. More details of the dynamics and the properties of these types of patterns are given in the next subsection. In addition, for protocol P1, we find (a) wave propagation without EAD formation (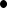) and (b) wave propagation, in which the AP shows EADs, but with no sustained electrical activity (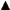). In protocol P2, we show that the typical non-EAD pattern is a stable, single spiral wave (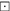), the light-gray SF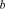 represents SF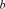-type patterns which terminated themselves during our simulations. The yellow, blue, and red colors indicate, respectively, no EAD, EAD, and oscillatory AP single-cell behaviors, as in Figure 4a.

We turn now to a characterization of the spatial patterns in our simulations.

### Characterization of the patterns

#### Spiral fibrillation type a

**Figure 9:**
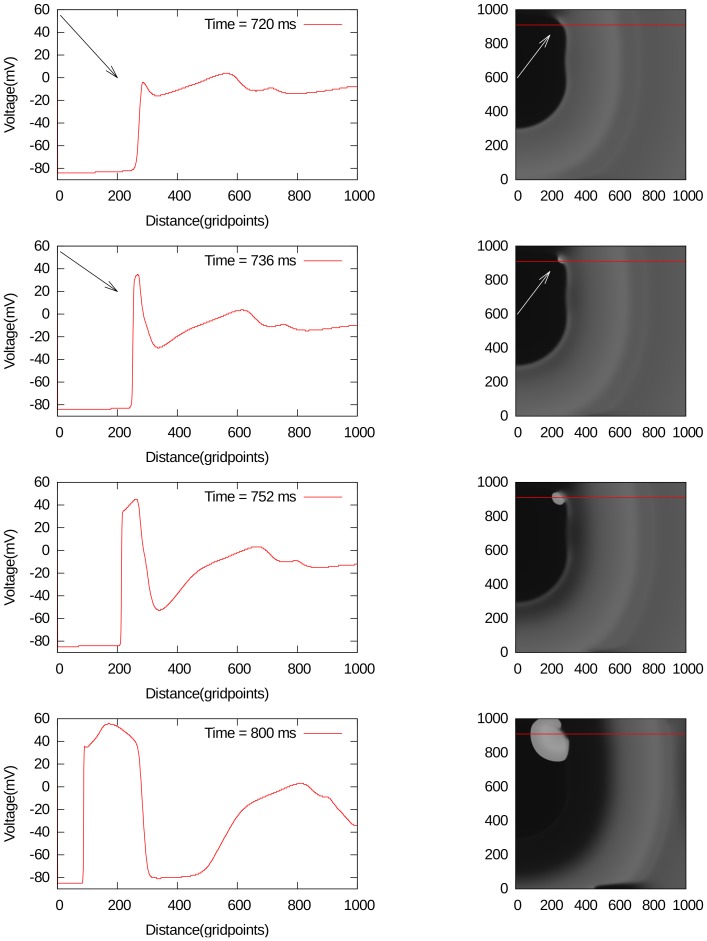
Typical backward break formation due to EADs. The left column- spatial pattern of voltage along the red line of 2D excitation pattern shown on the right. The parameter values are 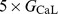, 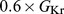.

In general, spiral fibrillation of type a consists of multiple interacting spiral waves. A spiral wave always occurs because of the formation of wave-breaks. The initiation of the first spiral, in protocol P2, is straightforward, as we create it by the S2 stimulus. Interestingly enough, we are also able to obtain initial breaks, after the point-stimulation protocol P1. We have found multiple ways by which this breaks occur. We show a few examples, by gray-scale plots of 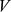, in Figure 9 and Figure 9 for different parameter values. In all these cases, only a single external stimulus is applied, and wave breaks occur either from local EADs, which has formed close to the boundary like in Figure 9, or from an EAD wave, which has split from the waveback and then again formed a wave that propagates forward 9. In order to show that this break is clearly due to EADs, we have shown how the voltage changes during these breaks in Figure 9 and Figure 9. In Figure 9, one can see how an EAD is formed around 720 ms (1st line, arrows), separates around 736 ms (second line, arrows) and creates a new stimulus in the backward direction between 752 and 800 ms (line 3 and 4). In Figure 9 a forward wave is created due to the formation of EADs. In the 1st line (480 ms), no EAD is clearly visible. However at 640 ms, an EAD is formed (2nd line) and separates around 720 ms (3rd line). Multiple wavebreaks in the forward direction are clearly seen around 800 ms (4th line).

**Figure 10:**
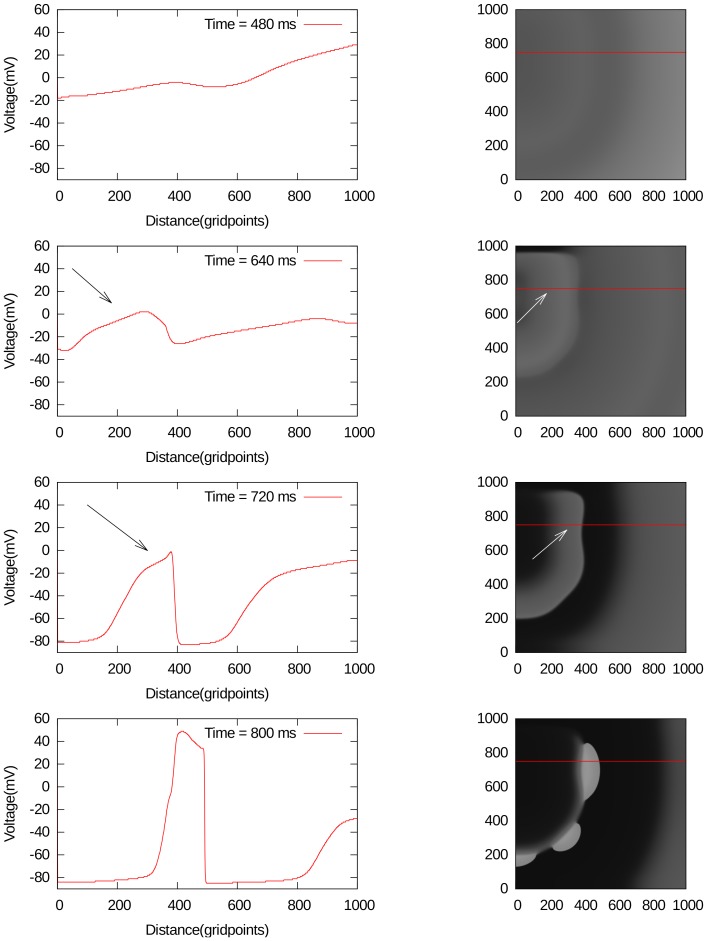
Typical forward break formation due to EADs. All notations the same as in Figure 9. The parameter values are 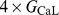, 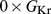.

In many cases, EAD waves do not result in the formation of an initial break. This explains why we do not have spiral fibrillation for all the parameter values in Figure 6a, as opposed to what we see in Figure 6b.

Although the first break can occur at different locations, the final state for all patterns, which we have obtained via protocol P1, is qualitatively similar. In Figure 7, we observe a second break of the wave, in the bottom of the simulation domain, for the parameter values 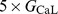 and 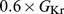 (Figure 7a). Subsequently, this wave keeps on breaking and we see that small spirals are formed (Figure 7b). Finally, a complex pattern emerges (Figure 7c–7e). This pattern consists of multiple, small, rotating spirals and is reminiscent of the complex excitation patterns that are usually observed after the process of spiral breakup [33], [34]. These patterns appear to be very chaotic because of the large number of interacting spirals and waves. Moreover, we do not see clear black regions separating the waves, which indicates the small amplitude of the action potentials that we characterize and discuss later. We call this pattern spiral fibrillation of type a (see also video S1). Notice, however, that the protocol P1 does not always result in such a pattern. In many cases, the initial wave break does not occur for a given initial condition.

For the protocol P2, we get similar patterns. Moreover, when we compare patterns for the same parameter values for the protocols P1 and P2, we find that the final state does not depend sensitively on the initial conditions (of course, for those conditions for which P1 leads to spiral fibrillation patterns of type a). We do see some differences in the patterns, when we use different parameter values. The closer our system is to the blue region in Figure 6, the more stable and larger are the spirals. By contrast, in the nearer the system is to the yellow region, the more chaotic is the spiral pattern, which now displays many small spirals.

Finally, notice that these types of patterns corresponds roughly to the single-cell behavior in which the AP shows an EAD. This spiral pattern of type a is the only type of spiral pattern that we have been able to obtain with protocol P1. Protocol P2 allows us to obtain a second type of spiral fibrillation, which we discuss in the next subsection.

#### Spiral fibrillation type b

**Figure 11:**
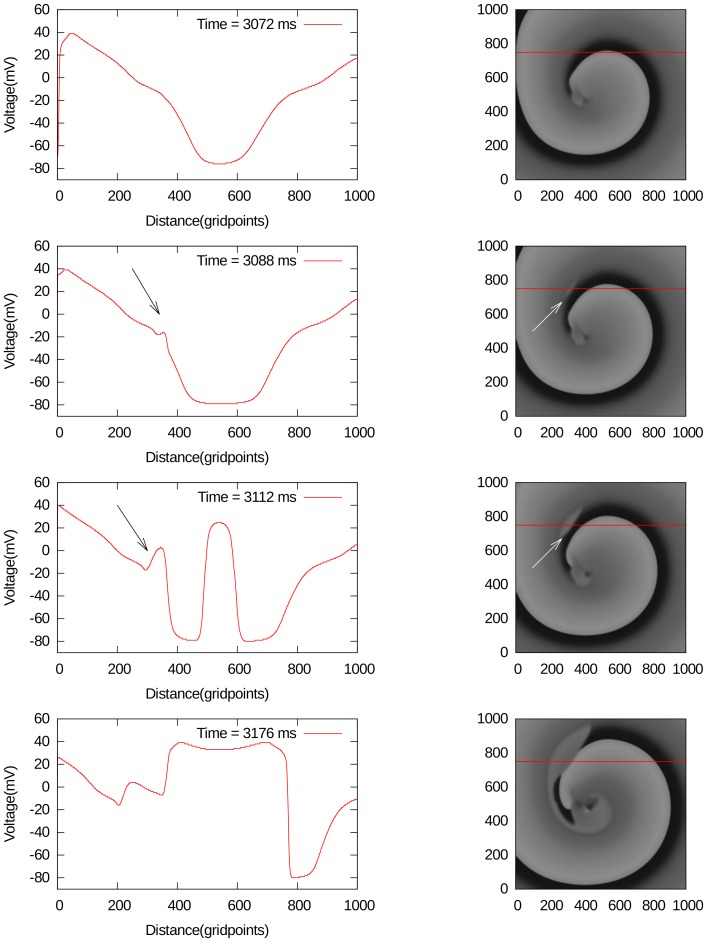
Typical break formation due to EADs in P2 protocol. All notations the same as in Figure 9. The parameter values are 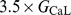, 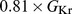.

We illustrate spiral fibrillation of type b by the representative gray-scale plots of 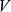 in Figure 8 (see also video S2). In Figure 8a we depict the spiral induced by the protocol P2. First, in frame 8b, we see that break up occurs close to the core of the spiral wave. Again, in order to show that this break is clearly due to EADs, we have shown how the voltage changes during these breaks in Figure 11. types of spiral fibrillation proceeds gradually. High in the yellow region of Figure 6, we observe spiral fibrillation of type b, whereas, in the vicinity of the blue region, we see the emergence of spiral fibrillation of type a. Notice that this pattern exists in the parameter region in which a single stimulus, in a single-cell, does not gave rise to an AP with an EAD.

#### Oscillatory fibrillation

**Figure 12:**
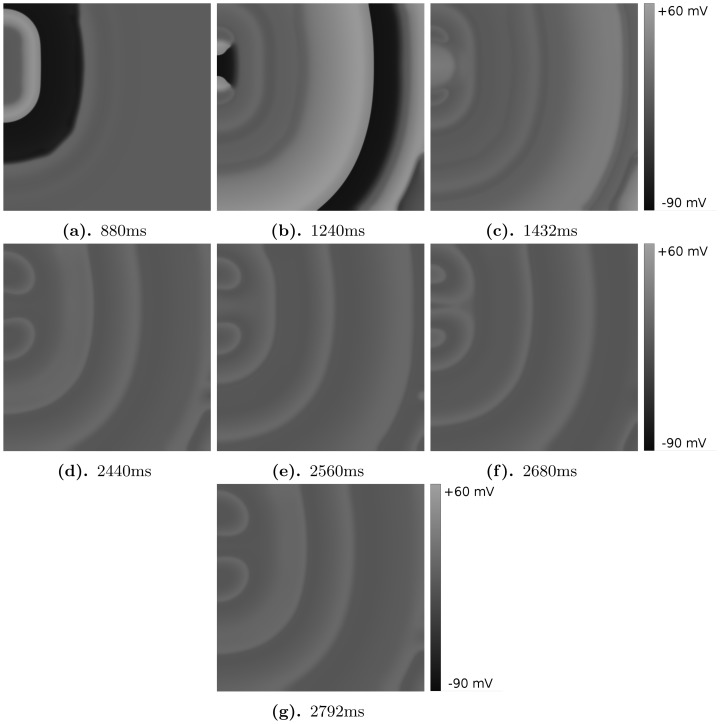
Focal type of fibrillation. Protocol P1 is used with the following parameters: 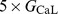, 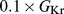.

We show another type of pattern in Figure 12, which demonstrates that, after the initial stimulus, the forward wave has split into two waves (see Figure 12a). After 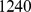 ms we see the formation of two breaks in the wave back (Figure 12b). However, these wave breaks do not form spirals, but yield two point sources (see Figures 12c – 12g), which then persist in the simulation domain. We refer to this as an oscillatory pattern (see also video S3).

**Figure 13:**
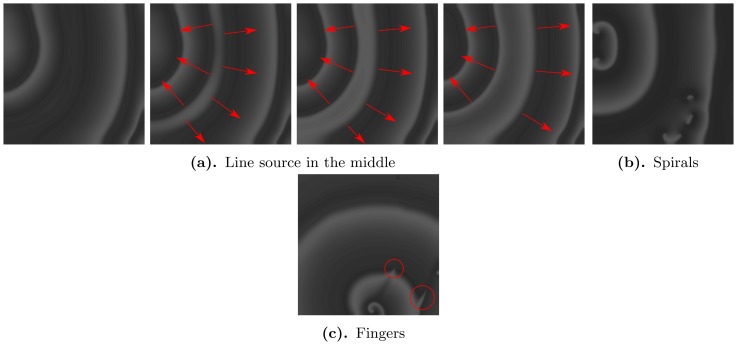
Illustration of different effects in the oscillatory patterns. (a) A line source with the parameter values 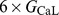 and 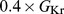, in the frames for 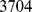 ms, 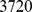 ms, 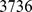 ms, 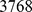 ms, (b) spirals for the parameter values 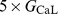 and 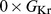, in the frame for 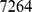 ms, (c) fingers for the parameter values 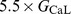 and 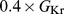, in the frame for 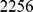 ms. In comparison with Figure 12, we have increased the contrast of all the frames to enhance the visibility of all the features in these gray-scale plots of V.

The patterns in this oscillatory regime have various unusual manifestations, e.g., in addition to point sources, lines of point sources can also emerge. In Figure 13a, we show how, in the middle of the simulation domain, a line of point sources generates two wavefronts that propagate in opposite directions. Spirals can also appear in this regime, e.g. in Figure 13b, we can distinguish 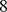 different spirals. Even though these spirals may look similar to the ones we have shown earlier, they are engendered by completely different mechanism as we discuss in the next subsection. We also find that (a) spirals can evolve into point sources and (b) we can have a type of fingering instability (Figure 13c), which is normally associated with a negative eikonal-curvature relation [35]. As we show in Section “Phase waves versus regular waves”0, all these patterns still have an oscillatory underlying dynamics.

In summary, then, we have found the following three different types of spatial patterns: (a) spiral fibrillation of type a, (b) spiral fibrillation of type b, and (c) oscillatory fibrillation. Other wave patterns, shown in Figure 6, represent patterns with EAD or no EAD waves without the formation of a sustained pattern or a single spiral, which is just the initial state that follows from the protocol P2. We now characterize the spiral and oscillatory patterns in a more quantitative way.

#### Rate dependence of the patterns

**Figure 14:**
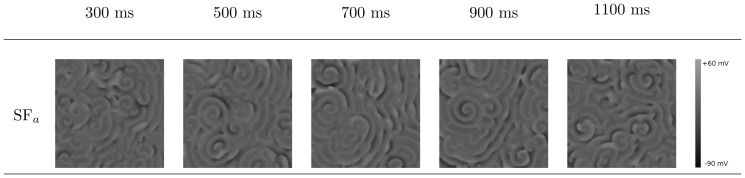
Spatial pattern of excitation developed after initial stimulation of the tissue with various frequencies (shown at the top of the figure). We used protocol P1 for the parameters: 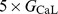, 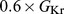. Pictures are taken at 10 s ).

The initial conditions P1 represents one of the standard conditions that can be used in numerical simulations; these conditions are easily reproducible; and they can be viewed as a limiting case of low-frequency stimulation of cardiac tissue and thus can be related to arrhythmias that occur at low heart rates. However, it is also interesting to see if similar phenomena can occur at other frequencies, as arrhythmias can occur at various heart rates. We have, therefore, used the stimulation protocol P1 again; but during the simulation, we have applied P1 with a certain rate ranging from 300 ms to 1000 ms. Figure 14 shows spatial patterns of excitation that develop after initial stimulation with different rates. We see that the final patterns are qualitatively the same in all cases, when we compare them with Figure 7. Therefore, we conclude that these patterns do not depdend sensitively on the initial conditions and frequency of stimulation.

#### L- type Calcium-mediated and Sodium-mediated waves

**Figure 15:**
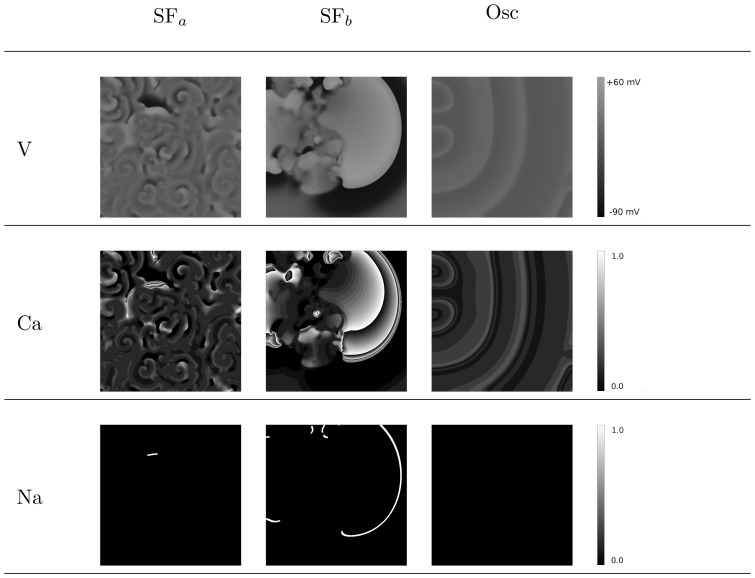
Final states for the different patterns with voltage (up), opening of Ca-gates (middle), and opening of sodium gates (down). If the gates are fully opened, the value on the picture is equal to 1 (white), if the gates are closes, the value is 0 (black). Ca-waves dominate in spiral fibrillation type a (Protocol: P1, parameters: 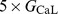, 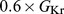, time: 9600 ms.), while mostly sodium mediated waves precede most of the waves in spiral fibrillation type b (Protocol: P2, parameters: 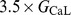, 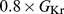, time: 9600 ms). For oscillatory fibrillation Ca-current induce the voltage oscillations of each cell (Protocol P1, Parameters: 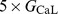, 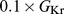, time: 2800 ms).

In normal conditions, excitation waves in cardiac tissue are driven by Na currents. However, in some situations, waves driven by Calcium can also occur [28]. To find out which of these types of waves we have in our systems, we plot, on the left of Figure 15, the spatial distribution of the voltage, in the middle, the opening and closing of the L-type Calcium gates (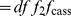 in Equation (3)), and, on the right, the opening of the Sodium gates (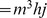 in Eq. (7)). We see that, for the first pattern, i.e., spiral fibrillation of type a (SF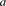), the final state is almost completely maintained by L type Calcium currents. The initial wave and the first breakup of the waves shown in Figs. 9 and 9, respectively, are induced by a Sodium current (not shown here). However, during the development of break up, we see more and more Calcium-driven waves and, eventually, the Na-current-driven waves disappear. For spiral fibrillation of type b (SF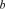), however, many waves, even in the final state, are induced by Sodium currents. This observation gives us a clear way of distinguishing between these two different types of spiral patterns. Moreover, the deeper the parameter values are located into the yellow region (further away from the red region) in Figure 6b, the more waves are induced by Sodium currents. We find that, for oscillatory fibrillation, the waves are always induced by Calcium, as we can see in the last frames in Figure 15 (Osc). For the three patterns, see the videos S4, S5 and S6.

#### Typical AP of a single-cell in the 2D tissue, ECG and Fourier transforms

**Figure 16:**
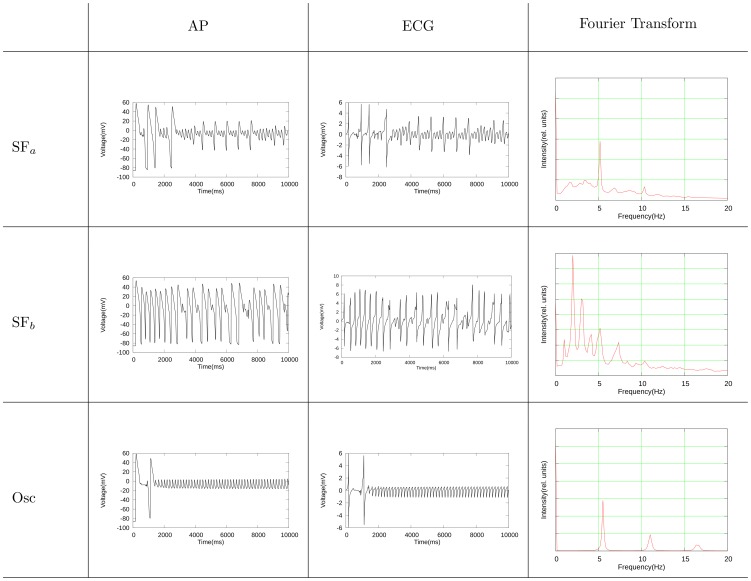
The AP, ECG, and temporal Fourier Transforms of 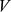 for the three different patterns. We show plots for the spiral fibrillation of type a (protocol P1 and the parameters 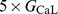 and 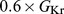), the spiral fibrillation type b (protocol P2 and the parameters 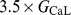 and 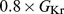), and the oscillatory fibrillation (protocol P1 and the parameters 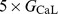 and 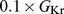).

We now contrast several characteristics of the three main types of patterns we have mentioned above. In particular, we compare the shapes of the APs in the middle of our simulation domains, the electrocardiogram (ECG) generated by a pattern, and finally the temporal Fourier Transform of 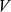 in the entire medium. The ECG's are calculated in the following way:
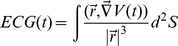
(9)where 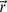 is a vector starting from the middle of the surface at perpendicular distance of 2.5 mm and pointing to the surface, 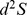 represents the surface we are studying and 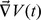 is the gradient of the corresponding point at the surface. The Fourier transforms are calculated for 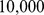 points that are distributed equally in our 2D domain. We then take the sum over all these points. The results are shown in Figure 16.

#### AP

For a spiral pattern of type a, a typical AP recording has 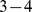 small pulses separated by a pulse of larger amplitude (Figure 16). We see that 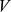 does not go below 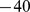 mV in such a recording. Thus, all upstrokes here are generated by the Ca current, as 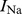 is inactivated if 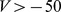 mV. It is interesting to note that we still observe two types of waves with different amplitudes (usually a few waves of small amplitude alternating with a wave that has a larger amplitude).

The AP shape for the SF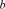 pattern is quite different from its counterpart for SF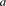. In the first 3 seconds, the former has no EADs in the AP. This is why SF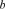 lies in the yellow region of Figure 4a. Indeed, in our single-cell simulations, we have only paced the cell once. This agrees with the first part of the SF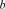 AP in Figure 16. Only after 3 seconds, the first EADs appear in the case of SF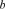 and the spiral breaks up, however, 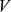 always goes back to a lower value than in an AP for SF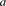, and, therefore, waves are still induced by 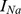.

For the oscillatory pattern (Osc), each cell exhibits a stable, low-amplitude oscillation (Figure 16). The amplitude of these oscillations increases as we change parameters to approach the boundary of the SF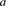 phase in Figure 4a. In Osc, the amplitude of these oscillation is small, so 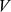 does not reach the threshold for inducing sodium mediated waves and, therefore, the oscillations are solely induced by L-type calcium currents.

#### Fourier Transform

It is interesting to characterize the pattern by the average of the temporal Fourier transform of the voltage 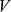. We obtain this by summing the temporal Fourier transforms of 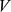 from its time series at 10000 points, which are evenly distributed through the simulation domain. In Figure 16 we portray the Fourier power spectrum, for the case SF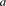. This has one maximum at a fundamental peak at 5.18 Hz (the inverse of 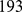 ms) for the P1 protocol and the parameters values 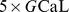 and 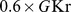. We find exactly the same peak with the P2 protocol. We conclude, therefore, that this maximum does not depend on the initial conditions. Furthermore, the period rotation of the spiral waves is given by the inverse of the frequency, i.e.,the Ca-spirals rotate once in 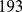 ms, which is also the average time period of the small oscillations in the AP.

**Figure 17:**
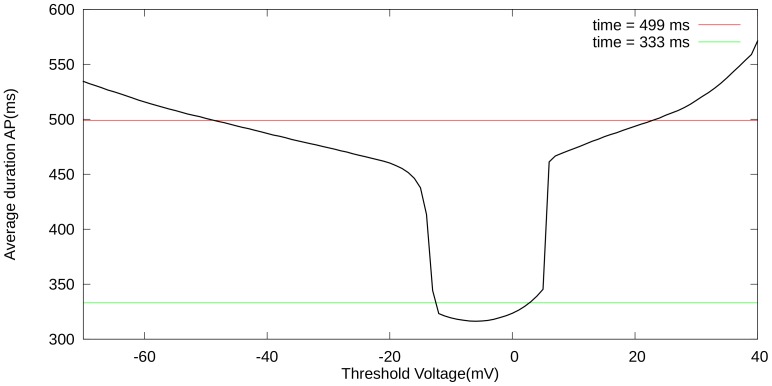
Sum of the power spectral densities of 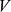. These power spectral densities are averaged over 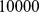 points in our simulation domain for the parameters 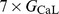 and 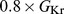, and the P2 or S1-S2 protocol (see Figure 6b).

We show in Figure 16 an illustrative plot of the power spectrum of 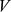 in the parameter regime where we get SF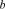 patterns. We obtain two principal peaks at 2 Hz (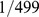 ms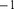) and at 3 Hz (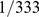 ms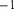) and several smaller peaks. Figure 17 shows that these peaks are connected to the interbeat intervals of waves of different amplitude. We see in Figure 17 that, if we measure the interbeat intervals for the same 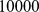 points for which we have calculated the power spectrum, then we see a longer interbeat intervals 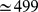 ms, for waves with amplitudes between 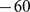 and 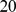 mV. We obtain shorter interbeat intervals 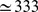 ms, for waves amplitudes between 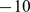 and 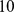 mV. However, the relation of these interbeat intervals to spiral-wave patterns is non-trivial as we do not have clear spiral waves here.

Figure 16 also shows the power spectrum of 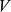 for the oscillatory pattern. This has a sharp peak at 6 Hz (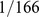 ms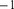), which is exactly the inverse of the oscillation period of the pattern in the AP. The closer the parameter values are to the boundary of SF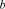 in Fig. 6b, the larger are the periods of the oscillations. The period of these oscillations is independent of how the oscillations are initiated. In particular, this period does not depend on whether we use the P1 or P2 protocols.

#### ECG

Finally, we plot the ECGs for each type of the excitation pattern. For SF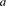 and SF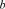 the ECG resembles voltage recordings and has some similarity with the ECG during TdP. For the oscillatory pattern, we see a stable periodic ECG.

#### Phase waves versus regular waves

We now illustrate another feature of our patterns. This is related to wave propagation and the distinction between real and phase waves. This requires some explanation as it has not been used widely in studies of wave propagation in cardiac tissue. By real waves we mean conventional electrical-activation waves, which arise because of an interplay of the excitability of the medium and the diffusion. Such waves travel with a velocity that is proportional to the square root of the diffusion constant. They are absorbed at impermeable boundaries and do not go through regions in which the medium is in a refractory state [36]. In contrast, phase waves, which are pseudo-traveling waves that occur often in oscillatory media [36], do not depend on the diffusion constant and are not hindered by impermeable boundaries. In real waves in cardiac-tissue models, local currents, generated at the upstroke of the wave, depolarize tissue in front of them. This enables wave propagation, however, we can also have phase waves without local interactions. For example, if we consider oscillators spread through space but uncoupled to each other, we can induce a pattern, which looks like a propagating wave, just by making a constant shift of phase between neighboring oscillators, as in a Mexican wave. Although this spatial pattern then looks like a wave, it is not a real wave as an impermeable barrier between two points does not block 'wave propagation' here. In this subsection we demonstrate that (a) the wave patterns in SF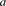 and SF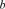 are, indeed, real waves, whereas (b) we have phase waves in the Osc state.

**Figure 18:**
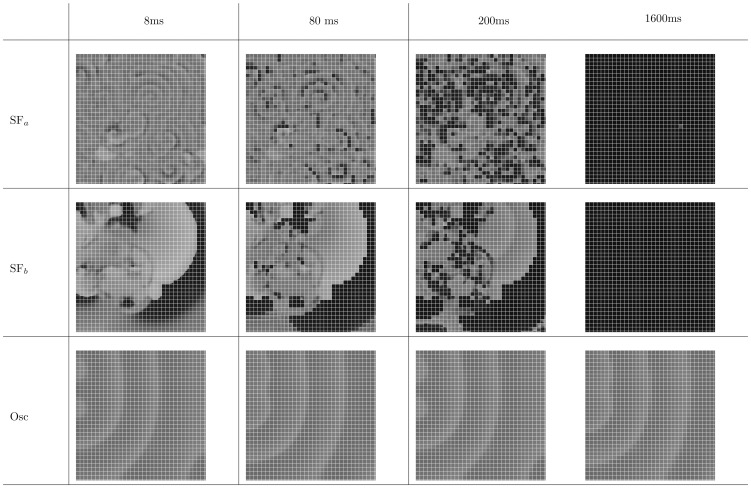
Illustration of phase wave activity in focal fibrillation type. Our simulation is divided (see text) into blocks of 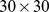 unit cells. The first frame is taken after 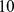 s for all patterns. The patterns have the following parameters. Spiral fibrillation type a: protocol P1 and parameters 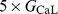 and 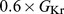, spiral fibrillation type b: protocol P2 and parameters 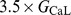 and 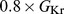, oscillatory fibrillation, protocol P1, and parameters 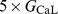 and 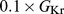.

To do this we use barriers, which are organized into a grid that is a square lattice. It has been shown that, if such a mesh is stimulated by an external current that makes it a refractory barrier, it removes wave patterns that are generated by real waves [37], [38]. Our grid subdivides our simulation domain into square unit cells of size 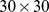. We prepare barriers in our domain, by stimulating the edges of our square unit cells, after the patterns shown in section 0 have developed. The wave patterns after 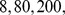 and 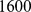 ms are shown, via gray-scale plots of 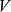, in Figure 18. The square-mesh barriers are clearly visible. We see that both spiral patterns, SF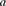 and SF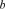, disappear after the barrier is imposed. This shows that they are, indeed, generated by real waves. However, the imposition of such a barrier, does not affect an oscillatory pattern, in which case we can still see the propagation of wave-like patterns. This shows clearly that the patterns we observe here are formed by phase waves, and they are a consequence mainly of the initial conditions formed during the formation of this pattern and not of real wave propagation. See also video S7, S8, and S9 to clearify Figure 18.

## Discussion

In this paper we have presented a comprehensive numerical study of 2D wave patterns, which are generated by cells that produce EAD responses. In our study we have used a state-of-the-art mathematical model for human ventricular cells [29], [30], and we have obtained EADs by increasing or modifying I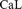, by decreasing I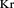 or I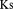, or by increasing I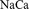. The main aim of the paper has been to find out how single-cells, which exhibit EADs, give rise to 2D patterns and fibrillation and then to classify these 2D patterns. We have not focused on a particular disease, but on more general properties of excitation patterns which should be common for any situation when the repolarization reserve of the cell is reduced. Our aim has been to link the single-cell behavior to different types of 2D excitation patterns.

It is well established that changes, similar to those used in our modeling studies, also promote the onset of EADs in experimental and clinical studies. An increase of I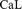 is one of the main effects of adrenergic stimulation, which promotes EADs [19]. Furthermore, isoproterenol (adrenergic agonist) and Bay K 8644 (calcium-channel agonist) are used to evoke EADs in experiments [39]–[43]. Second, a blockage of I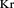 and/or I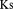 by drugs, such as E-4031, Quinidine (Quinidine also affect other channels besides I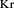 and I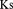.) has been shown to promote EADs in experiments [44]. A reduction of I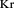 or I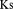 can also occur as a result of mutations in several forms of the long-QT syndrome [45], [46]. Arrhythmias in that cases are also associated with the onset of EADs [3], [24], [47]–[49]. It has also been suggested that I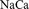 is important for the onset of EADs, especially during adrenergic stimulation [10]. All these changes are also in line with a general theory of EAD onset (decreasing of the repolarization reserve) by increasing the inward currents and decreasing the outwards currents [3].

We have shown that the mechanism of EAD generation, in our model, is the reactivation of I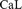, which is also common in many types of EAD generation [10], [19]. In many cases, EAD generation in our model is facilitated by secondary CICR and subsequent I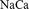 activity. However, this is not strictly necessary for the generation of an EAD, as we have also seen examples without secondary CICR. It has also been shown that 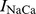 plays a role in the formation of the EAD as, e.g., in Refs. [10], [11], [14]. However, in our study, I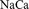 does not directly contribute to the formation of the EAD, it only plays a role afterwards: if a secondary release of Ca from the SR has elevated the Ca concentration in the cytoplasm, and Ca has to be removed again, then 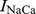 increases in the inward direction, but, at that moment, the AP is repolarizing again.

At the cellular level we have obtained phase diagrams in two-dimensional parameter spaces, that show the parameter ranges in which EAD activity occurs (we use parameters such as I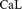, I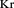, I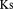, or I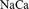). We have also classified such activity. In all the cases we have studied, a decrease of the repolarization reserve results in transitions from a normal AP to AP with a single EAD, then to multiple EADs, and finally to oscillatory or triggered activity. This is in agreement with the studies of Ref. [31] for the LR1 model. Thus, our study confirms that the effects of such changes are (a) model independent, (b) are generic properties of mathematical models for cardiac cells, and (c) do not depend on the specific parameters which we alter to obtain EADs. It is important here to decrease the repolarization reserve progressively.

We have then generated 2D patterns, which occur in our simulation domain with cells that show EADs and obtained therefrom phase diagrams, in two-dimensional parameter spaces, that help us to find the regions of stability of these patterns and leads to a natural classification for them. We have shown that all non-trivial spatial patterns can be classified into 3 main classes: spiral wave fibrillation, of types a and b, and oscillatory spatial patterns. The tissue size used in our simulations is rather large, as we want to study the wave dynamics with minimal effects of boundaries. However, we have also performed some simulations on patches with smaller sizes (5×5 cm) and we observed patterns of the same type for the same parameter values as for the tissue of larger size in most of our calculations. We have also studied the onset of the wave breaks in 2D. We show in Figures 7, 8 and 10 that these breaks arise clearly from EADs. Wave breaks can occur at different locations and in the forward (Figure 7) as well as in the backward (Figure 8) directions. However, much more research can be done in the study of the mechanism of initiation of the wave breaks and its relation to the electrothonic load, such as boundary conditions, anisotropies, etc. In addition, as EADs can be generated by many different mechanisms [10], it would be interesting to check if all our results can be reproduced for other mechanisms as well. We think that our results will hold, as the most important phenomenon, for all these EAD mechanisms is a reduction of the repolarization reserve. However, this requires additional study.

Parameters for which we obtain oscillatory patterns are located in the range for which we find oscillatory patterns in our single-cell simulations. We demonstrate that (a) spiral fibrillation of type a occurs normally in the parameter region with a single or multiple EADs and (b) spiral fibrillation of type b is observed at the boundary between the no-EAD and EAD regions and also in the region where a single stimulus in the single-cell does not show any EADs. Note that there is no clear boundary between these two latter regimes: we observe a smooth, gradual transition from one regime to another.

To the best of our knowledge, our study is the first one that has obtained phase diagrams, in two-dimensional parameter spaces, which lead to regions of stability for the different spatial patterns we find in our simulation domain that consists of EAD cells. However, a few examples of complex spatial patterns, originating from EADs, have been reported in Refs. [26]–[28], [50]. A direct comparison of these patterns with those in our study is difficult, because most of the cited works do not study specifically the nature of wave propagation in their systems (Ca or Na). Two exceptions are the studies of Ref. [28] and [26]. However, in the former case, only the initial phases of break up have been shown. In [26], a multiple spiral wave pattern, similar to the one shown in Figure 7, has been shown (see Fig.3 from [26]). In Ref. [27], the break up arises because of a substantial regional prolongation of refractoriness in a spatially heterogeneous medium, which is different from our homogeneous simulations.

We find that spiral patterns of type b and oscillatory patterns are mediated by Ca waves, whereas spiral type a patterns are formed by Sodium mediated waves. In the case of oscillatory patterns, we have observed phase waves. As far as we aware, we have given the first demonstration of the existence of phase waves in a detailed model of cardiac tissue. Our study also suggests a clear method for identifying phase waves in such systems. This can be used in experimental research too. In our method we investigate wave propagation in the presence of oscillatory EADs. We demonstrate that phase waves cannot be eliminated by propagation barriers. We note that phase waves do not propagate because of local interactions of adjacent cells. Such behavior in single cells has also been found in other modeling studies (see, e.g., [31]). In addition, the experimental study of reference [51], has shown examples of triggered activity because of EADs in a culture of cardiac cells representing either hypertrophic or fibrotic cardiac tissue. The red parts of Figures 2B and 2C of reference [51] also show regions of tissue that do not repolarize any more and a region with small-amplitude variations of the transmembrane potential. However, it remains unclear if the observed wave patterns are generated by oscillatory sources similar to those studied in our models. Overall, much more work needs to be done in order to show that such regimes occur in real cardiac tissue.

The effects, which we have found, can be studied experimentally. The most relevant experimental setup is a cell culture of the type used in Ref. [52]. In particular, one can use a cell culture of neonatal myocytes, which are engineered in a 2D patch, and has been shown suitable for studying wave propagation. As in our simulations, I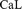 can be increased by the use of certain drugs, such as isoproterenol, and I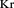 can be decreased by e.g. E-4031 and Quinidine. We expect that, progressively increasing the L -type calcium current and decreasing I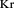 or I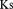, will go through the three different regimes: first spiral fibrillation type b, then spiral fibrillation type a and finally oscillatory fibrillation. For the study of SF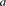 and SF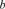, one can use optical voltage mapping to distinguish clearly between the two patterns as done in [28]. Some addition information could come from optical mapping of calcium transient and its comparison with voltage. In addition, by using the new technique of human pluripotent stem cells [53], [54], one could even test this with human cardiac tissue.

Finally, let us consider some limitations of this study. We have only studied a monodomain idealized, homogeneous and isotropic tissue model in 2D. Generally, studies on EAD dynamics have usually been carried out in 2D tissue [14], [25]–[28]. Only in Ref. [14], [50] have the authors provided a few examples of 3D and anatomically realistic simulations. However, as the main aim of our manuscript has been to investigate VF dynamics in a two-dimensional parametric space, which requires around 600 simulations for the different parameter values, we have preferred to use a 2D simulation domain. We expect that 3D or anatomically realistic domains will not change the type or mechanism of VF, but might increase the complexity of the patterns. Preliminary results indeed show that in the whole heart, EADs give rise to complex fibrillation patterns. The organization of real cardiac tissue is much more complex than the 2D homogeneous, isotropic tissue studied here [15]–[18], [55]. It is widely expected that heterogeneities, anisotropy, and other characteristics of cardiac tissue influence the onset and complexity of EAD patterns. Specific effects of these factors will therefore be investigated in another study. However, the understanding of these new phenomena can be based on the results of our simulations, in a homogeneous domain. This is because heterogeneity and other factors bring an additional layer of complexity on top of the effects that we have studied here. Also, we have not used a bidomain model mathematical model for our simulations. However, we expect that, even if we use a bidomain version of the our mathematical model, our results will not change qualitatively. For example, a recent study [56] has shown that, if we do not have large electrical stimuli, the differences between mono-domain and bidomain mathematical models for cardiac tissue are not substantial. We have only studied this in one particular human cardiac cell model [30]. It would therefore be interesting to see if the same effects are also present in other models of human cardiac cells, such as those of Refs. [57], [58].

## Supporting Information

Figure S1Important ionic currents related with EAD formation. We show all the currents which directly contribute to the potential when no EAD is present (green line with parameters: 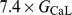, 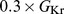) and when an EAD is present (red line parameters: 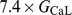, 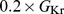).(TIFF)

Figure S2Important ionic currents related with EAD formation part 2. Parameters left: 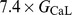, 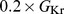, parameters middle: 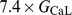, 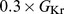, parameters right: 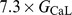, 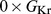.(TIFF)

Video S1An example of a spiral fibrillation of type a. The parameters are 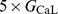, 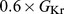, initiating with protocol P1. See also Figure 7.(AVI)

Video S2An example of a spiral fibrillation of type b. The parameters are 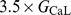, 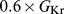, initiating with protocol P2. See also Figure 8.(AVI)

Video S3An example of a spiral fibrillation of type b. The parameters are 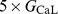, 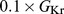, initiating with protocol P1. See also Figure 12.(AVI)

Video S4This video shows voltage (left), opening of Ca-gates (middle), and opening of sodium gates (right) in the SF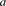 pattern. The parameters are 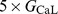, 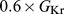, initiating with protocol P1. See also Figure 15. In the beginning of the movie, Sodium waves are still present, however, when the SF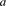 pattern emerges, it is clearly maintained by L type Calcium currents.(AVI)

Video S5This video shows Voltage voltage (left), opening of Ca-gates (middle), and opening of sodium gates (right) in the SF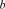 pattern. The parameters are 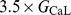, 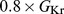, initiating with protocol P2. See also Figure 15. The SF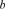 pattern is maintained by Sodium mediated waves.(AVI)

Video S6This video shows Voltage voltage (left), opening of Ca-gates (middle), and opening of sodium gates (right) in the oscillatory pattern. The parameters are 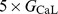, 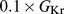, initiating with protocol P1. See also Figure 15. The oscillatory pattern is clearly maintained by L type Calcium currents.(AVI)

Video S7Starting from the pattern spiral fibrillation of type a (with parameters 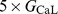, 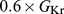 and initiated with protocol P1), we have divided the medium into blocks of 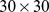 unit cells. The barriers are not conducting. The pattern does not survive the division of the medium, and the spirals disappear. See also Figure 18.(AVI)

Video S8Starting from the pattern spiral fibrillation of type b (with parameters 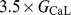, 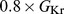 and initiated with protocol P2), we have divided the medium into blocks of 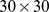 unit cells. The barriers are not conducting. The pattern does not survive the division of the medium, and the pattern disappears. See also Figure 18.(AVI)

Video S9Starting from the pattern oscillatory fibrillation (with parameters 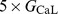, 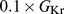 and initiated with protocol P1), we have divided the medium into blocks of 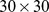 unit cells. The barriers are not conducting. The pattern is not much affected by the division of the medium. This proves that the waves are in fact phase waves. See also Figure 18.(AVI)
