# Correction: A Study of Early Afterdepolarizations in a Model for Human Ventricular Tissue

**DOI:** 10.1371/annotation/ebef014a-20cf-4ebb-a074-84239532f1d0

**Published:** 2014-01-22

**Authors:** Nele Vandersickel, Ivan V. Kazbanov, Anita Nuitermans, Louis D. Weise, Rahul Pandit, Alexander V. Panfilov

In the Funding statement, a funding organization and grant providing financial support for the fourth author is incorrectly omitted.

The following should be read with the Funding statement: 

"Louis D Weise acknowledges support from the Netherlands Organization for Scientific Research (http://www.nwo.nl) [^] (NWO Grant No. 613.000.604) of the research Council for Physical Sciences (EW)."

