# Supplementary figures and images for: A Study of Early Afterdepolarizations in a Model for Human Ventricular Tissue
Source: PLoS One. 2014 Jan 10;9(1):e84595. doi: 10.1371/journal.pone.0084595 (PMC3888406; doi:10.1371/journal.pone.0084595)

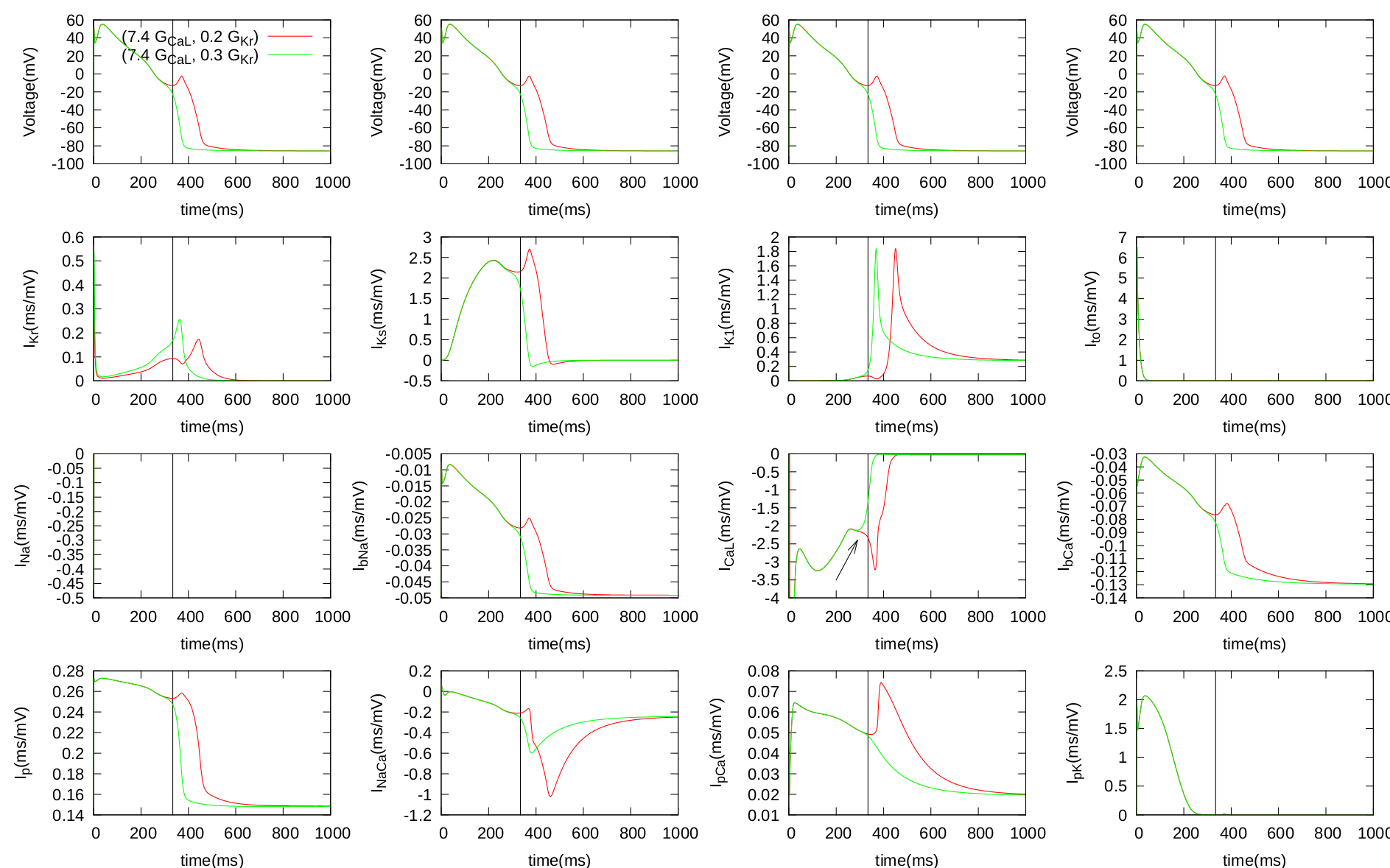

Supplement: Figure S1 — Important ionic currents related with EAD formation. We show all the currents which directly contribute to the potential when no EAD is present (green line with parameters: , ) and when an EAD is present (red line parameters: , ). (TIFF) [file pone.0084595.s001.tiff]

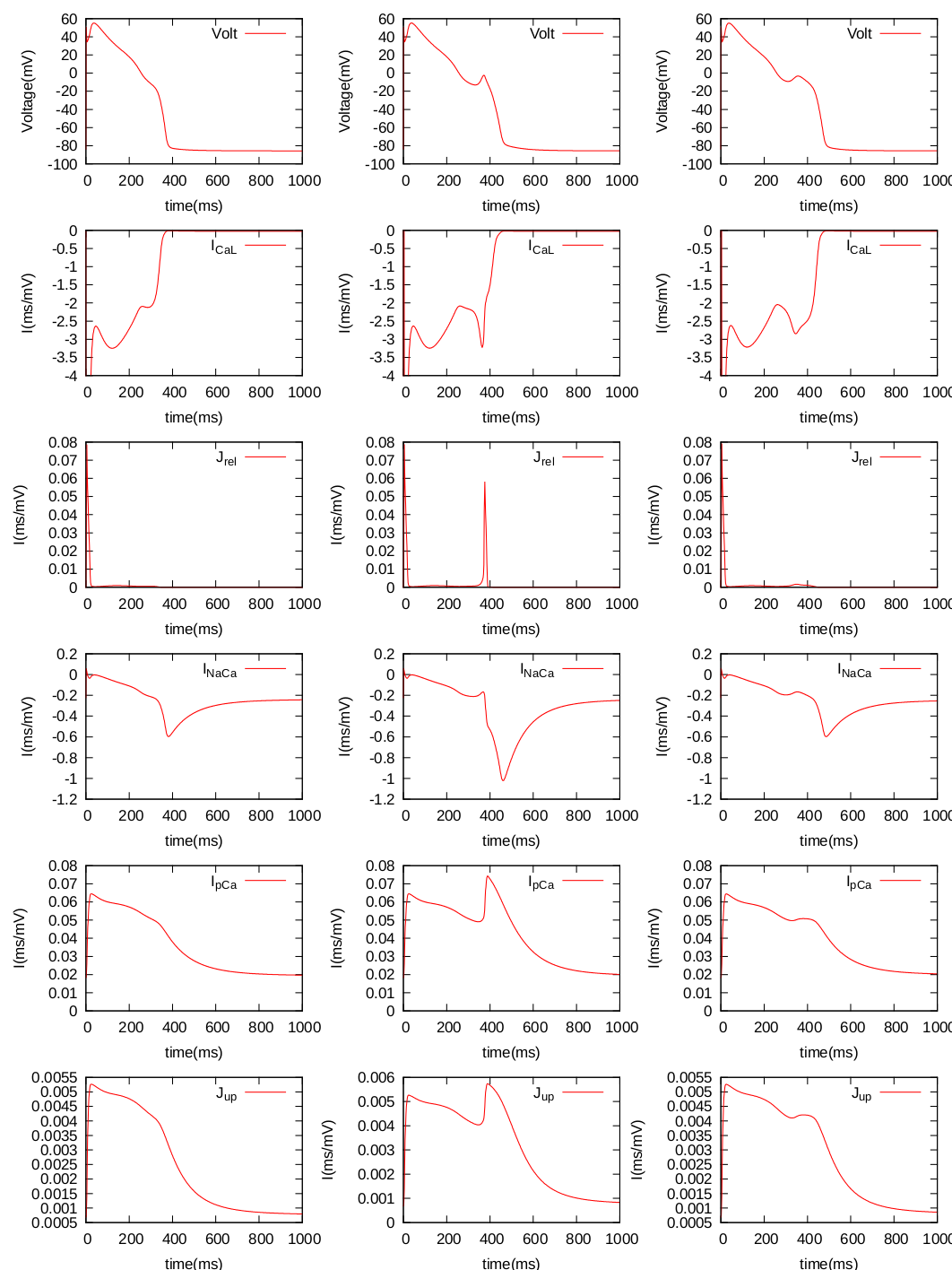

Supplement: Figure S2 — Important ionic currents related with EAD formation part 2. Parameters left: , , parameters middle: , , parameters right: , . (TIFF) [file pone.0084595.s002.tiff]
